# The relation between oxytocin receptor gene polymorphisms, adult attachment and Instagram sociability: An exploratory analysis

**DOI:** 10.1016/j.heliyon.2021.e07894

**Published:** 2021-09-22

**Authors:** Alessandro Carollo, Andrea Bonassi, Ilaria Cataldo, Giulio Gabrieli, Moses Tandiono, Jia Nee Foo, Bruno Lepri, Gianluca Esposito

**Affiliations:** a Department of Psychology and Cognitive Science, University of Trento, Rovereto, Italy; b Mobile and Social Computing Lab, Foundation Bruno Kessler, Trento, Italy; c Psychology Program, School of Social Sciences, Nanyang Technological University, Singapore, Singapore; d Lee Kong Chian School of Medicine, Nanyang Technological University, Singapore, Singapore; e Human Genetics, Genome Institute of Singapore, Singapore, Singapore

**Keywords:** Gene*Environment, Adult attachment, Close relationship, Oxytocin Receptor Gene, rs53576, rs2254298, Online behavior, Social media, Social network, Instagram

## Abstract

Oxytocin is a primary neuropeptide which coordinates affiliative behavior. Previous researchers pointed to the association between genetic vulnerability on *Oxytocin Receptor Gene* (*OXTR*) and environmental factors (e.g., social relationships) to comprehend social behavior. Although an extensive knowledge of in-person social interactions has been obtained, little is known about online sociability. A gene-environment perspective is adopted to examine how *OXTR* and adult attachment moderate Instagram behavior. The genetic factors within the regions *OXTR*/rs53576 (A/A homozygotes vs G-carriers) and *OXTR*/rs2254298 (G/G homozygotes vs A-carriers) were assessed. The Experience in Close Relationships-Revised (ECR-R) questionnaire was used to collect participants' (N = 57, 16 males) attachment with a partner. The number of posts, followed people (“followings”) and followers were obtained from Instagram, and the Social Desirability Index (SDI) was calculated as the ratio of followers to followings. Interaction effects between *OXTR* groups and ECR-R scores on the number of posts and SDI were hypothesized. Results showed an effect of rs53576 on the number of Instagram followings. Specifically, people with A/A *OXTR*/rs53576 genotype had more followings than G-carriers independent of the anxiety or avoidance felt towards their partner. These preliminary results offer insights into future investigations on social media behavior.

## 1 Introduction

People forge social bonds throughout their life, from the first sight of their primary caregiver to the affection felt for their partner. Social attitudes not only have a re-creative value, but they are also crucial for the development of social and life skills. In the human life course, socialization's origins have been traced back to the interplay between genes and environment.

### 1.1 The role of the oxytocin receptor gene on socialization

Social behavior finds its origins in the biological roots of the individual starting from the genetic predispositions. In the field of behavioral genetics on sociability, some authors have focused on the genes that rule the levels of oxytocin regulated by the *Oxytocin Receptor Gene* (*OXTR*). Generally, oxytocin is a neuropeptide that plays a crucial role for social skills in mammals [1], [2], [3], [4], [5], [6], [7]. Oxytocin has a primary effect on the parental motivation towards the offspring: oxytocin plasma levels in mothers and fathers are correlated with positive engagement in parent-infant interactions consequently influencing the infant's socio-emotional development [8], [9], [10]. As candidate substrates of early and long term epigenetic changes and mediators of caregiving affect social behavior throughout human development [11], [12], oxytocin receptors are mapped in brain areas that are typically involved in reproductive, social and adaptive behaviors, such as the limbic system and the brainstem [13], [14]. Higher oxytocin levels could reinforce social experiences by causing a reduction in the levels of anxiety [15], [16]. Numerous *OXTR* Single-Nucleotide Polymorphisms (SNPs), such as *OXTR*/rs53576 and *OXTR*/rs2254298, have been shown to predict parenting behavior and attachment [17], [18], [19], [20], [21]. For instance, the G allele of *OXTR*/rs53576 is associated with increased parental responsiveness [22], whereas the same allele on *OXTR*/rs2254298 is linked to lower plasma oxytocin and to less parental touch [23].

Different theoretical frameworks have been proposed to provide an explication of sociability under a gene*environment perspective. In the intersection of hereditary factors and environmental triggers which shape human development, the *Social Salience Hypothesis of oxytocin* [24], [25] attributes distinctive social behavioral patterns associated with different *OXTR* alleles to the perceived cues of a given social environment [26], [27]. An intriguing view is also offered by the *Differential Susceptibility model* [28], [29], which stipulates that the susceptibility to the environment depends on the so-called “plasticity genes”. These genes (e.g., *OXTR*) lead to broader individual plasticity. In fact, given a specific allele, individuals would not only show amplified adverse effects when exposed to a negative environment [30], [31], but also they would experience greater benefit from a positive and healthy environments [32], [33].

The two distinct *OXTR*/rs53576 and *OXTR*/rs2254298 SNPs appear to be favorable candidates when looking for the genetic modulation of social behavior and cognition [34], [35]. Specifically, a given allele within each of the two genetic regions could confer a peculiar level of vulnerability or susceptibility, either in an adaptive or in a maladaptive direction, to the environment in the individual [36].

### 1.2 Environmental influences on socialization and their interplay with the oxytocin receptor gene

Among multiple factors that shape sociability, environmental influences such as familiar education, parental bonding and adult attachment play a role in regulating in-person and online sociability [37], [38], [39], [40], [41]. According to the prototypical hypothesis of attachment, the relational patterns with peers and the partner are influenced by the early relationships with parents during infancy and childhood, which appear to be stable throughout life [42], [43], [44], [45]. Nevertheless, the revisionist hypothesis points to the evidence that the adult relationships may alter the early attachment representations and form new attachment models based on the novel social experiences [46]. Similarly, the quality of adult relationships with peers and one's partner affects different domains of life. In fact, people from high-quality marriages (i.e., people who show better marital adjustment and higher marital satisfaction) manifest better physical and mental health than the ones in low-quality marriages [47], [48], [49], [50], [51], [52]. Within the context of gene-environment interactions, multiple studies focused on social behavior as a product of a differential genetic sensitivity (i.e., *OXTR*/rs53576; *OXTR*/rs2254298) to positive and negative environments, such as successful or dysfunctional relationships with a partner. Previous works reported that people with the G allele of the *OXTR*/rs53576 are more sensitive to hostile environments seeking higher social support when compared to the A allele [53], [54]. Individuals carrying G/G genotype or having a partner with G/G genotype also reported higher marital satisfaction than A-carriers [55]. With regards to *OXTR*/rs2254298, the debate on the role of vulnerability genes is still open. In fact, if on one side G/G genotype has been observed to be highly associated with separation anxiety and depression [56], on the other, females G/G homozygotes showed less attachment anxiety than A-carriers, while male G/G homozygotes exhibited less autism-associated traits than A-carriers [57]. The majority of studies consider A/A genotype as the one conferring the vulnerability to the environment when adopting the *Differential Susceptibility Model* [58], [59].

However, there is a paucity in research investigating online sociability, particularly those considering the continuous and reciprocal interaction between the environment to which a person is exposed and their genetic profile [60], [61], [62].

### 1.3 Socialization in the context of social networks sites

Nowadays, socialization takes place both offline and online, persuading researchers to examine not only the in-person social interactions but also virtual social interactions. The last decade has observed an increasing number of studies inspecting numerous social networks sites (SNSs), such as Instagram, Facebook, Twitter, whose centrality in human life has grown impressively [63], [64]. These digital environments have become the theaters of novel and interactive social behaviors, instantly shared with large communities from all over the world.

Among the SNSs, Instagram proved to be a useful platform to explore online sociability [65], [66]. Here, users connect by posting pictures and daily stories, following other users' profiles and chatting with them actively. The studies investigating online sociability on Instagram have detected both positive (e.g. decreased feelings of solitude or increased motivation and inspiration) [67], [68] and negative effects, usually linked to poorer mental health outcomes on users [69], [70], [71].

Although there is a growing number of studies on SNSs such as Instagram, it is not yet clear whether in-person and online social interactions share the same mechanisms. To fill this existing gap in the literature, the current paper aims to adopt a gene*environment perspective to investigate Instagram sociability. Specifically, the current exploratory research focuses on Instagram sociability as a result of potential interaction between genetic factors (i.e. *Oxytocin Receptor Gene*) and the environmental effects related to the quality of adult relationships with their partners (i.e. adult attachment with an intimate partner). Specifically, two *OXTR* SNPs (*OXTR*/rs53576 and *OXTR*/rs2254298) were selected as genetic factors. The Experience in Close Relationships-Revised (ECR-R) questionnaire was used to assess the individual's self-perception of adult attachment with an intimate partner [72]. Three Instagram variables were collected: 1) the number of posts, 2) the number of followed people (here called “followings”) and the number of followers. An index, called “Social Desirability Index” (SDI), was computed as the ratio of followers to followings. Higher values of the SDI would indicate greater online sociability.

In line with the *Differential Susceptibility model*, an interaction effect between the genetic factors and the close relationship scores was hypothesized for each Instagram variable. Specifically, regardless of gender, we formulated one hypothesis: *OXTR*/rs2254298 A-carriers and *OXTR*/rs53576 G-carriers would show a higher number of posts and values of SDI when involved in a positive and favorable relationship with their partner (lower scores in the ECR-R dimensions Avoidance and Anxiety) compared to *OXTR*/rs2254298 G/G homozygotes and *OXTR*/rs53576 A/A homozygotes. Conversely, *OXTR*/rs2254298 A-carriers and *OXTR*/rs53576 G-carriers would show a lower number of posts and SDI when involved in a negative and unfavorable relationship with their partner (higher scores in the ECR-R dimensions Avoidance and Anxiety) compared to *OXTR*/rs2254298 G/G homozygotes and *OXTR*/rs53576 A/A homozygotes.

## 2 Methods

### 2.1 Participants

Sixty-one (N = 61) non-parent adults were recruited among students enrolled at the Nanyang Technological University (Singapore). Participants of the study were all Singaporean. Exclusion criteria were: (i) present or lifetime history of psychiatric, neurological or genetic disorders, (ii) not having an Instagram account, and (iii) being older than 30 years. This third criterion was adopted in order to get a pool of comparable data from participants in the same age range. It is to note that this criterion did not affect the distribution of genetic and attachment data, and reflected anyway the majority of the examined university population. We could not take into account data from four (N = 4) participants because of failures in either the online compilation of questionnaires or in Instagram data extraction. In the end, our final sample consisted of 57 adults (16 males) between 18 and 25 years-old (*M* = 20.89, *SD* = 1.59; Males: *M* = 22.56, *SD* = 1.15; Females: *M* = 20.24, *SD* = 1.24).

### 2.2 Data collection

The methods and analytic plan for this study were preregistered on the Open Science Framework at the following address: https://osf.io/t78fu. Genetic, behavioral and Instagram data were collected. The research was authorized by the Ethical Committee of Nanyang Technological University (IRB-2015-08-020-01). Informed consent was obtained from all participants. The study followed the Declaration of Helsinki. The genetic assessment was conducted on anonymized samples at the Nanyang Technological University (Singapore). Instagram data and questionnaires' data were anonymized at the beginning of the data collection. With regards to genetic data, a sterile cotton swab for genotyping was used to collect a buccal mucosa sample of DNA from each participant. Concerning the behavioral assessment, every participant completed online the self-report questionnaire Experience in Close Relationships-Revised (ECR-R). To collect Instagram indexes, an in-house built Python-based web-scraper developed on BeautifulSoup, a python package designed for web-scraping [73], was employed. Firstly, the algorithm extracted the content of participants' Instagram profile from the DOM of the page. Subsequently, the algorithm detected the specific objects — namely “posts”, “followers”, and “following” — in order to limit the amount of personal information accessed from each profile. A manual inspection of the data was then conducted to check for the presence of missing values due to technical errors, which were then manually updated (e.g. connection timeout, server errors). The same script was employed in previous works [60].

The final dataset generated for this work is available online on the Data Repository of the Nanyang Technological University (DR-NTU Data) [74].

### 2.3 Genetic assessment

The genetic assessment of the present study employed the method reported by Bonassi et al. [75]. DNA extraction and genotyping were performed by ACGT, Inc. (Wheeling, IL). DNA was extracted by using Oragene DNA purification reagent and its concentrations were evaluated through spectroscopy (NanoDrop Technologies, USA). Each DNA sample was increased by polymerase chain reaction (PCR) for the *OXTR* gene rs53576 region target with the primers 5-GCC CAC CAT GCT CTC CAC ATC-3 and 5-GCT GGA CTC AGG AGG AAT AGG GAC-3. A PCR reaction of 20 ll, comprising 1.5 ll of genomic DNA from the test sample, PCR buffer, 1 mM each of the forward and reverse primers, 10 mM deoxyribonucleotides, KapaTaq polymerase, and 50 mM MgCl2 was executed. PCR operation comprised of 15 minute denaturation at 95 ^∘^C, and 35 cycles at 94 ^∘^C (30 s), 60 ^∘^C (60 s), 72 ^∘^C (60 s) and a final 10 minute step at 72 ^∘^C. PCR reactions were genotyped with an ABI 3730xl Genetic Analyzer (Applied Biosystems Inc.) and normalized with GeneScan 600 LIZ (Applied Biosystems, Inc.) size standards on each sample. Genotypic data were inspected using GeneMapper ID (Applied Biosystems, Inc.).

The same procedure was used to assess the *OXTR* gene rs2254298 region. However, the forward and reverse primers were instead 5-TGA AAG CAG AGG TTG TGT GGA CAG G-3 and 5-AAC GCC CAC CCC AGT TTC TTC-3 respectively.

### 2.4 Close relationships

In order to assess the participants' relationship with their partner, the Experience in Close Relationship-Revised questionnaire was used. Developed by Fraley et al. [76], the ECR-R (average Cronbach's α=0.89) is a 36-item self-report questionnaire on a 7-point response scale, that examines the close relationship across two dimensions: Anxiety (Cronbach's α=0.93) and Avoidance (Cronbach's α=0.85). While the former is usually linked to worries about the relationship, and fear of rejection from one's partner (i.e., “I find that my partner(s) don't want to get as close as I would like”), the latter mostly refers to discomfort with intimacy and closeness to one's partner (i.e., “I find it difficult to allow myself to depend on romantic partners”). The current instrument has obtained extensive approval, high reliability and adequate consistency in appraising the constructs related to the attachment styles with the romantic partner [77], [78], [79]. Several studies applied the ECR-R to understand how the adult attachment security can be modulated by infant attachment, childhood maltreatment and can moderate social cognition, emotional regulation, personality and mindfulness [80], [81], [82], [83]. The ECR-R has also been used by researchers in the field of behavioral genetics. In this context, the ECR-R dimensions were conceived as potential environmental variables able to affect physiological and psychological variables [84], or the product of gene*environment interactions [85], [86]. We calculated the scores for the two constructs of anxiety and avoidance by following the scoring indications reported by Picardi et al. [87].

### 2.5 Instagram variables

Four variables were considered as highly representative of online sociability on Instagram. Number of posts indicates the number of content published by a participant's profile. On Instagram, the number of posts is equivalent to the number of published pictures, since this social network allows accounts to publish pictures and video with an auxiliary text. The literature regarding the determinants of one's number of posts shows contrasting results. For instance, depressed mood can be an antecedent of an increased posting activity as well as narcissism, or even extraversion [88], [89], [90]. Therefore, an increased activity on SNSs is not a mere positive or negative indicator of the user's well-being, but may depend on diverse and even contrasting drives (e.g., the need to escape from reality, the need to get in touch with others, and so on). The number of followed people (“followings”) is an index that reflects the number of profiles that a participant follows. On Instagram, following allows the user (the follower) to view — or ask to view, in the case of private profiles — other users' posts (the “followings”) in the general feed. Conversely, the number of followers is defined by the number of profiles that follows the considered participant. Broadly speaking, publishing a post can be considered as an active form of social communication, for the user decides what others can see from their profile. Following, instead, is a type of passive communication, for the user is exposed to the contents selected by others. Additionally, the SDI was computed as the ratio of followers to followings. This parameter, previously adopted as a measure of general sociability [60], [61], estimates the balance between the number of followers and followings for each account. By simultaneously taking into account the amount of profiles that one follows or is followed by, the SDI can furnish an estimate of the centrality of a specific user within the network of contacts. SDI can theoretically range from 0 to infinite, where 0 indicates a strong interest on following other people's contents and infinite indicates a scarce interest on following other accounts together with a strong appeal for other people. Values close to 1 for SDI would indicate a balanced activity in the network, ideally with the same values of followers and followings.

### 2.6 Statistical analysis

Statistical analysis and data visualization were performed using R (R-core base version 4.0.0.). Instagram data were standardized using z-score transformation and visually inspected for normality. To reduce the variability of the data, and to increase the statistical power of performed tests [91], [92], outliers — defined as values that differ by two or more standard deviations from the mean of the distribution — were inspected in the database. Overall, 8 data point were identified as outliers (3 for followings number, 2 each for posts number and SDI, 1 for follower number).

Before and after the outliers removal, normality of ECR-R and Instagram scores were tested by computing the values of skewness and kurtosis, and their distributions were manually inspected by visualization via boxplots, density plots, and quantile-quantile plots. Of the investigated variables, when all the data points were analyzed, the participants' number of posts, SDI and number of followers were not distributed in a Gaussian fashion. When outliers were removed from the dataset, only the participant's number of posts did not follow the Gaussian distribution. Thus, the related samplings were adjusted and log-transformed. Subsequently, the assumption of the homogeneity of variance was verified.

To detect potential gene*environment interactions on Instagram variables considered for the two hypotheses (number of posts, SDI values), two different mixed ANCOVAs were employed for each *OXTR* gene SNP: one ANCOVA with Instagram number of posts and one with Instagram SDI as DV for *OXTR*/rs53576, whereas one ANCOVA with Instagram number of posts and one with Instagram SDI as DV for *OXTR*/rs2254298 [59], [93], [94]. To take into account the increased possibility of committing a type I error, a false discovery rate correction was employed by means of a Bonferroni Correction (corrected α=0.025). Bonferroni's method for multiple testing correction was chosen rather than the available alternative approaches to apply a more conservative correction.

A prior sensitivity power analysis conducted on G*Power [95] (power = 0.80) estimated that the analysis could detect a medium effect size (f = 0.42) via analysis of covariance given the current sample size (*N* = 57) and the corrected α=0.025 (ANCOVA: fixed effects, main effects and interactions).

In the analysis, *OXTR*/rs53576 (A/A vs. G-carriers) and *OXTR*/rs2254298 (G/G vs. A-carriers) were considered as separate predictors in distinct analyses. Two out of four mixed ANCOVAs included the *OXTR* gene genotype rs53576 (A/A and G-carriers) as a between-subject factor and the ECR-R dimensions (Anxiety and Avoidance) as continuous covariates, as in Equation (1).

(1) $${\text{Instagram}}_{\text{variable}}=\text{OXTR SNP}⁎({\text{ECR}}_{\text{Anxiety}}+{\text{ECR}}_{\text{Avoidance}})$$

Likewise, the two remaining ANCOVAs included the *OXTR* gene genotype rs2254298 (G/G and A-carriers) as the between-subject factor, and the ECR-R dimensions (Anxiety and Avoidance) as continuous covariates, as in Equation (1). If any significant difference emerged on the values of the Instagram variables in relation to the participants' gender, gender was included in the ANCOVA equation as a between-subject factor.

## 3 Results

### 3.1 Results observed on unaltered data

#### 3.1.1 Preliminary analysis on the genetic variables

As common practice in the genetic studies, in order to have statistically comparable groups of participants, the heterozygotes and less numerous homozygotes groups were merged together in a group of “allele-carriers” [94]. Therefore, within the *OXTR*/rs53576 DNA region, participants with at least one G allele (G/G homozygotes or A/G) were categorized into a single G-carriers group. The distribution in our sample was 39% for A/A homozygous and 61% for G-carriers. The frequencies of the genotype were: A/A = 22 (38.60%), A/G = 30 (52.63%), G/G = 5 (8.77%), and the distribution was consistent with the Hardy-Weinberg Equilibrium ($X^{2}$ (1) = 1.38, *p* = 0.241, ns). For the *OXTR* gene rs2254298 region, participants with at least one A allele (A/A homozygotes or G/A) were classified into a single A-carriers group. The distribution in our sample was 56% for G/G homozygotes and 44% for A-carriers. The frequencies of the genotype were: A/A = 4 (7.02%), G/A = 21 (36.84%), G/G = 32 (56.14%), and the distribution was consistent with the Hardy-Weinberg Equilibrium ($X^{2}$ (1) = 0.05, *p* = 0.828, ns). Table 1 reports the observed frequency of *OXTR*/rs53576 and *OXTR*/rs2254298 genotypes by the total sample size. In the context of *OXTR*/rs53576, participants gender ($X^{2}$ (1) = 0.17, *p* = 0.683, ns) did not significantly differ between the two groups A/A vs G. As regards *OXTR*/rs2254298, participants' gender ($X^{2}$ (1) = 0.81, *p* = 0.367, ns) did not significantly differ between the two groups G/G vs A.

**Table 1:** Frequency values of the total sample in Oxytocin Receptor Gene (*OXTR*) rs53576 and Oxytocin Receptor Gene (*OXTR*) rs2254298 genotypes. The frequency percentage of each genotype is reported between parenthesis. The presented allelic frequencies are consistent with the Hardy-Weinberg equilibrium (for *OXTR*/rs53576, *X*^2^ (1) = 1.38, *p* = 0.241, ns; for *OXTR*/rs2254298, *X*^2^ (1) = 0.05, *p* = 0.828, ns). The present data refer to the 57 participants which completed the entire assessment across three levels: genetic (*OXTR*/rs53576; *OXTR*/rs2254298), behavioral (Experience in Close Relationships-Revised questionnaire) and Instagram (number of posts, following, followers, and the Social Desirability Index) data collections.

*OXTR*/rs53576	
Alleles	Total
AA	22 (38.60%)
AG	30 (52.63%)
GG	5 (8.77%)
G-carriers	35 (61.40%)
Total	57

*OXTR*/rs2254298	

Alleles	Total
AA	4 (7.02%)
GA	21 (36.84%)
GG	32 (56.14%)
A-carriers	25 (43.86%)
Total	57

#### 3.1.2 Preliminary analysis on ECR-R and Instagram variables

Table 2 describes the values concerning the data distribution of each continuous variable (two ECR-R dimensions and four Instagram variables) in terms of skewness, kurtosis and descriptive statistics (see “Statistical Analysis” for further details).

**Table 2:** Summary of the descriptive statistics for each continuous variable. The distribution of each ECR-R (Experience in Close Relationships-Revised) dimension and Instagram variable is described in terms of Skewness, Kurtosis, Minimum (Min), first Quartile (1st Q), Median, Mean, third Quartile (3rd Q), Maximum (Max) and Standard Deviation (SD). Instagram variables were standardized using z-score transformation and inspected for normality. Skewness and Kurtosis values were computed for each continuous subscale of the ECR-R questionnaire and each Instagram variable. Participants' number of posts, Social Desirability Index, and number of followers did not follow the Gaussian distribution, and were thus adjusted by log-transformation.

Variable	Skewness	Kurtosis	Min	1st Q	Median	Mean	3rd Q	Max	SD
Anxiety	-0.38	-0.02	1.39	3.33	3.94	3.94	4.56	6.22	1.08
Avoidance	-0.15	-0.39	1.06	2.11	2.94	2.95	3.67	5.39	0.93
Posts number	3.43	12.61	-0.60	-0.51	-0.36	0.00	0.04	4.61	1.00
Log-transformed posts number	2.08	4.78	0.33	0.40	0.49	0.62	0.71	1.89	0.34
Social Desirability Index	5.66	35.39	-1.16	-0.27	-0.12	0.00	0.01	6.76	1.00
Log-transformed Social Desirability Index	2.28	12.73	-0.17	0.55	0.63	0.64	0.70	2.17	0.29
Followings	0.53	-0.46	-1.53	-0.77	-0.12	0.00	0.67	2.47	1.00
Followers	4.74	27.70	-0.80	-0.50	-0.17	0.00	0.24	6.40	1.00
Log-transformed followers number	1.71	5.84	0.18	0.40	0.61	0.63	0.81	2.13	0.33

To exclude any significant effect of participants' gender on Instagram variables, four preliminary two-tailed Student's *t*-tests were performed (corrected α=0.0125). As expected, no significant differences in the standardized Instagram number of posts (*t* = -0.16, df = 55, *p* = 0.874), 95% CI [-0.22, 0.18], followings (*t* = 2.20 df = 55, *p* = 0.032), 95% CI [0.06, 1.20], and on the standardized SDI (*t* = 1.60, df = 55, *p* = 0.116), 95% CI [-0.03, 0.31] were found between male and female participants. Conversely, a significant difference between genders was detected for Instagram number of followers (*t* = 2.60, df = 55, *p* = 0.012), 95% CI [0.05, 0.42]. This evidence allows to analyze data of female and male participants together in the subsequent analysis on Instagram number of posts, followings, and SDI. As for Instagram number of followers, participants' gender was included in the subsequent analysis and examined as a potential moderator factor.

#### 3.1.3 Hypothesis-driven analysis: Instagram number of posts and SDI

Means and standard error means of Instagram number of posts and SDI for *OXTR*/rs53576 and *OXTR*/rs2254298 are reported in Table 3.

**Table 3:** Mean values in Oxytocin Receptor Gene (*OXTR*) rs53576 A/A homozygotes and G-carriers and Oxytocin Receptor Gene (*OXTR*) rs2254298 G/G homozygotes and A-carriers divided by Experience in Close Relationships-Revised (ECR-R) dimensions (high or low) on Instagram number of posts and Social Desirability Index. Standard error means are reported between parentheses. Instagram behavior is reported by users' *OXTR*/rs53576 phenotype and ECR-R dimensions, consistently with the literature on the topic and a previous publication [60].

*OXTR*/rs53576				

Number of posts				

ECR-R dimension	Low/AA	Low/G	High/AA	High/G
Anxiety	0.76 (0.12)	0.54 (0.05)	0.67 (0.15)	0.58 (0.07)
Avoidance	0.91 (0.15)	0.62 (0.06)	0.49 (0.04)	0.50 (0.06)

Social Desirability Index				

ECR-R dimension	Low/AA	Low/G	High/AA	High/G
Anxiety	0.59 (0.03)	0.74 (0.11)	0.63 (0.02)	0.58 (0.07)
Avoidance	0.62 (0.02)	0.66 (0.07)	0.59 (0.02)	0.66 (0.11)

*OXTR*/rs2254298				

Number of posts				

ECR-R dimension	Low/GG	Low/A	High/GG	High/A
Anxiety	0.60 (0.06)	0.67 (0.13)	0.67 (0.11)	0.54 (0.07)
Avoidance	0.74 (0.09)	0.74 (0.12)	0.52 (0.05)	0.48 (0.06)

Social Desirability Index				

ECR-R dimension	Low/GG	Low/A	High/GG	High/A
Anxiety	0.69 (0.11)	0.65 (0.05)	0.62 (0.08)	0.58 (0.04)
Avoidance	0.66 (0.06)	0.62 (0.06)	0.65 (0.13)	0.60 (0.02)

**Figure 1:**
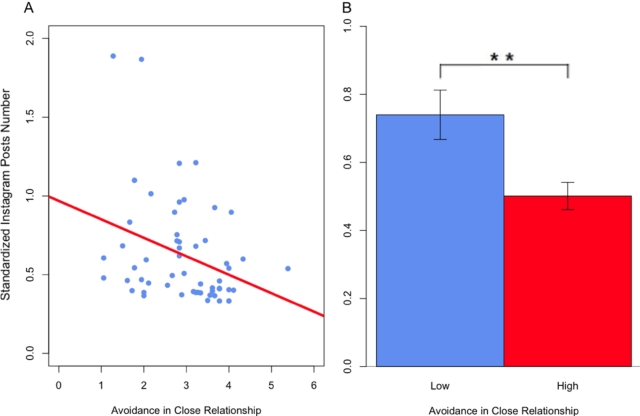
Main Effect of Avoidance in Close Relationships on Instagram Number of Posts. (**A**) Negative linear association between avoidance and Instagram number of posts. (**B**) Comparison between the number of posts in participants with low (blue) and high (red) avoidance in close relationships (** *p* < 0.01).

**Figure 2:**
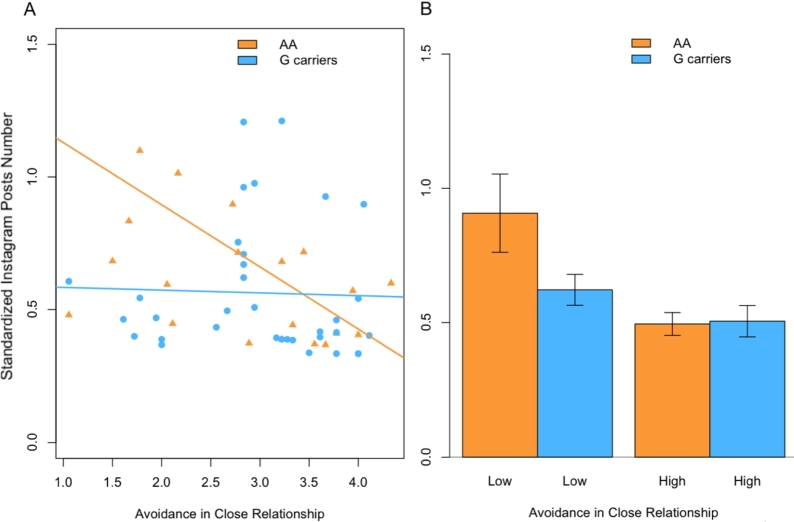
Interaction between Avoidance in Close Relationships and *OXTR*/rs53576 genotype on determining the Instagram number of posts. (**A**) Linear relationships between avoidance in close relationships and Instagram number of posts divided by genotype (in orange A/A homozygotes, in light blue C-carriers). (**B**) Comparison between the number of Instagram posts in participants with high and low levels of avoidance. Barplots are reported for both the genetic groups: A/A homozygotes in orange and C-carriers in light blue.

Results revealed no significant main effects of *OXTR*/rs53576 (*p* > 0.025) on Instagram number of posts and SDI. Nevertheless, a main effect emerged for ECR-R avoidance on the number of posts (*F*(1, 56) = 9.96, *p* < 0.003, p$\eta^{2}$ = 0.16; see Fig. 1). Moreover, a significant interaction between ECR-R avoidance and *OXTR*/rs53576 on Instagram number of posts was found (*F*(1, 56) = 7.12, *p* < 0.010, p$\eta^{2}$ = 0.12; see Fig. 2). No other main effect or interaction effect was detected on the number of posts and SDI (Table 4).

**Table 4:** Hypothesis-driven analysis: statistics' summary of ANCOVAs on Instagram number of posts and Social Desirability Index. For each Instagram variable considered in the hypothesis (Instagram number of posts and SDI) as dependent variable, one mixed ANCOVA was performed with *OXTR* gene genotype rs53576 (A/A and G-carriers) as a between-subject factor and the ECR-R dimensions (Anxiety and Avoidance) as continuous covariates (see the top section of the Table). For each Instagram variable, one mixed ANCOVA was also performed with the *OXTR* gene genotype rs2254298 (G/G and A-carriers) as a between-subject factor and the other parameters unchanged (see the below section of the Table). A significant main effects of the avoidance in close relationships was found on the number of posts. Also, avoidance in close relationships by interacting with the *OXTR*/rs53576 resulted to modulate the number of posts. (* *p* < 0.025; ** *p* < 0.001).

*OXTR*/rs53576					

Number of posts					

Variable	DF	Sum square	Mean square	*F* value	*p*-value

*OXTR*/rs53576	1	0.338	0.3381	3.925	0.05299
Anxiety	1	0.068	0.0676	0.785	0.37972
Avoidance	1	0.858	0.8579	9.958	0.00269 **
*OXTR*/rs53576: Anxiety	1	0.025	0.0248	0.287	0.59417
*OXTR*/rs53576: Avoidance	1	0.613	0.6130	7.116	0.01022 *
Residuals	51	4.394	0.0861		

Social desirability index					

Variable	DF	Sum square	Mean square	*F* value	*p*-value

*OXTR*/rs53576	1	0.035	0.03469	0.373	0.544
Anxiety	1	0.034	0.03417	0.367	0.547
Avoidance	1	0.000	0.00023	0.002	0.961
*OXTR*/rs53576: Anxiety	1	0.023	0.02330	0.250	0.619
*OXTR*/rs53576: Avoidance	1	0.004	0.00424	0.046	0.832
Residuals	51	4.748	0.09309		

*OXTR*/rs2254298					

Number of posts					

Variable	DF	Sum square	Mean square	*F* value	*p*-value

*OXTR*/rs2254298	1	0.014	0.0144	0.146	0.70366
Anxiety	1	0.034	0.0341	0.348	0.55801
Avoidance	1	1.017	1.0172	10.358	0.00224 **
*OXTR*/rs2254298: Anxiety	1	0.108	0.1075	1.095	0.30034
*OXTR*/rs2254298: Avoidance	1	0.113	0.1133	1.153	0.28792
Residuals	51	5.008	0.0982		

Social desirability index					

Variable	DF	Sum square	Mean square	*F* value	*p*-value

*OXTR*/rs2254298	1	0.027	0.02727	0.293	0.590
Anxiety	1	0.029	0.02887	0.311	0.580
Avoidance	1	0.001	0.00113	0.012	0.912
*OXTR*/rs2254298: Anxiety	1	0.026	0.02594	0.279	0.600
*OXTR*/rs2254298: Avoidance	1	0.022	0.02177	0.234	0.630
Residuals	51	4.739	0.09293		

Similarly, when considering *OXTR*/rs2254298, the same main effect of avoidance in close relationships on the number of posts on Instagram was detected (*F*(1, 56) = 10.36, *p* < 0.002, p$\eta^{2}$ = 0.17) (see Table 4; see Fig. 1).

To test the directionality of the effects found in the hypothesis-driven analyses, consequent post-hoc analyses were implemented. In particular, avoidance from the partner emerged to negatively predict one's number of posts on Instagram (*t*(55) = -2.54, *r* = -0.32, *p* = 0.014, 95% CI [-0.54, -0.07]; see Fig. 1A). In other words, the higher the avoidance, the lower the number of Instagram posts. Moreover, after applying the median split to the values of avoidance in close relationships, the result was confirmed by a two-tailed Student's *t* test. Specifically, a significant difference emerged when comparing participants with low and high values of avoidance in regards to their number of Instagram posts (*t*(55) = 2.85, *p* = 0.006, 95% CI [0.07, 0.41]) (see Fig. 1B).

Also the effect found for the interplay between the *OXTR*/rs53576 genotype and ECR-R avoidance on the number of posts was examined with post-hoc tests. In particular, after applying the median split procedure on the ECR-R avoidance, it emerged that the distribution of genotypes (G/G vs. A-carriers groups) was not significantly different between participants showing high rather than low avoidance ($X^{2}$ (1) = 0.03, *p* = 0.867, ns). Avoidance in close relationships resulted to be significantly correlated with the number of posts for the individual in the group of A/A carriers (*t* = -2.80, df = 20, *r* = -0.53, *p* = 0.011, 95% CI [-0.78, -0.14]). Conversely, the correlation between avoidance and number of posts on Instagram was not significant for the G-carriers (*t* = -0.22, df = 33, *r* = -0.04, *p* = 0.831, 95% CI [-0.37, 0.30]). Homogeneity of variance of the Instagram number of posts by avoidance in close relationships was checked (${\text{K}}^{2}$ = 9.55, df = 1, *p* = 0.002) (see Fig. 2).

#### 3.1.4 Exploratory analysis: Instagram number of followings and followers

Means and standard error means of Instagram number of followings and followers for *OXTR*/rs53576 and *OXTR*/rs2254298 are reported in Table 5.

**Table 5:** Mean values in Oxytocin Receptor Gene (*OXTR*) rs53576 A/A homozygotes and G-carriers and Oxytocin Receptor Gene (*OXTR*) rs2254298 G/G homozygotes and A-carriers divided by Experience in Close Relationships-Revised (ECR-R) dimensions (high or low) on Instagram number of followings and followers. Standard error means are indicated between parentheses.

*OXTR*/rs53576				

Number of followings				

ECR-R Dimension	Low/AA	Low/G	High/AA	High/G
Anxiety	0.78 (0.30)	-0.23 (0.19)	-0.08 (0.34)	-0.26 (0.21)
Avoidance	0.54 (0.31)	-0.04 (0.25)	0.21 (0.38)	-0.44 (0.14)

Number of followers				

ECR-R Dimension	Low/AA	Low/G	High/AA	High/G
Anxiety	0.76 (0.06)	0.67 (0.11)	0.59 (0.09)	0.52 (0.06)
Avoidance	0.72 (0.08)	0.62 (0.07)	0.62 (0.08)	0.57 (0.10)

*OXTR*/rs2254298				

Number of followings				

ECR-R Dimension	Low/GG	Low/A	High/GG	High/A
Anxiety	0.16 (0.27)	0.23 (0.27)	-0.21 (0.28)	-0.18 (0.23)
Avoidance	0.08 (0.26)	0.37 (0.31)	-0.12 (0.29)	-0.31 (0.16)

Number of followers				

ECR-R Dimension	Low/GG	Low/A	High/GG	High/A
Anxiety	0.72 (0.11)	0.68 (0.07)	0.54 (0.08)	0.55 (0.06)
Avoidance	0.63 (0.07)	0.70 (0.08)	0.64 (0.13)	0.53 (0.04)

The hypothesis-driven approach adopted for Instagram number of posts and SDI was extended as exploratory analysis to the number of followings and followers. Therefore, a Bonferroni's correction was separately applied for the two exploratory tests (corrected α=0.025). To identify exploratory gene*environment interactions on two further Instagram variables that were collected (number of followings and followers), two mixed ANCOVAs were employed for each *OXTR* gene SNP: one ANCOVA with Instagram's number of followings and one with Instagram number of followers as DV for *OXTR*/rs53576, whereas one ANCOVA with Instagram's number of followings and one with Instagram number of followers as DV for *OXTR*/rs2254298. For each ANCOVA the between-subject factor and the covariates were unchanged, as in Equation (1). Moreover, since Instagram number of followers emerged to be significantly different between genders, participants' gender itself was included as a moderator factor in the ANCOVA equations regarding the number of followers.

The ANCOVA performed on Instagram number of followings revealed a significant main effect of *OXTR*/rs53576 genotype (*F*(1, 56) = 6.19, *p* < 0.016, p$\eta^{2}$ = 0.11), as indicated in Table 6. No further significant interactions between the ECR-R subscales and *OXTR*/rs53576 on the Instagram number of followings were observed (see Table 6).

**Table 6:** Exploratory analysis: statistics' summary of ANCOVAs on Instagram number of followings and number of followers. For each Instagram variable considered in the exploratory analysis (Instagram number of followings and followers) as dependent variable (DV), one mixed ANCOVA was performed with *OXTR* gene genotype rs53576 (A/A and G-carriers) as a between-subject factor and the ECR-R dimensions (Anxiety and Avoidance) as continuous covariates, as in Equation (1) (see the top section of the Table). Gender was added as a further between-subjects factor in the analysis including Instagram number of followers. For each Instagram variable, one mixed ANCOVA was also performed with the *OXTR* gene genotype rs2254298 (G/G and A-carriers) as a between-subject factor and the other parameters unchanged (see the below section of the Table). A gender effect on the number of followings was confirmed, and a significant main effects of the genotype was found on the number of followings. (* *p* < 0.025).

*OXTR*/rs53576					

Number of followings					

Variable	DF	Sum square	Mean square	*F* value	*p*-value

*OXTR*/rs53576	1	5.50	5.505	6.193	0.0161 *
Anxiety	1	0.27	0.266	0.299	0.5870
Avoidance	1	1.72	1.717	1.932	0.1706
*OXTR*/rs53576: Anxiety	1	2.37	2.369	2.665	0.1088
*OXTR*/rs53576: Avoidance	1	0.81	0.809	0.910	0.3446
Residuals	51	45.33	0.889		

Number of followers					

Variable	DF	Sum square	Mean square	*F* value	*p*-value

*OXTR*/rs53576	1	0.095	0.0948	0.886	0.3515
Gender	1	0.712	0.7118	6.652	0.0132 *
Anxiety	1	0.132	0.1317	1.231	0.2731
Avoidance	1	0.020	0.0197	0.184	0.6699
*OXTR*/rs53576: Gender	1	0.049	0.0485	0.453	0.5042
*OXTR*/rs53576: Anxiety	1	0.166	0.1661	1.552	0.2192
*OXTR*/rs53576: Avoidance	1	0.002	0.0020	0.019	0.8921
Gender: Anxiety	1	0.008	0.0077	0.072	0.7892
Gender: Avoidance	1	0.008	0.0085	0.079	0.7795
*OXTR*/rs53576: Gender: Anxiety	1	0.003	0.0032	0.029	0.8644
*OXTR*/rs53576: Gender: Avoidance	1	0.018	0.0182	0.170	0.6821
Residuals	45	4.816	0.1070		

*OXTR*/rs2254298					

Number of followings					

Variable	DF	Sum square	Mean square	*F* value	*p*-value

*OXTR*/rs2254298	1	0.01	0.0138	0.014	0.908
Anxiety	1	0.61	0.6106	0.603	0.441
Avoidance	1	2.58	2.5842	2.553	0.116
*OXTR*/rs2254298: Anxiety	1	0.61	0.6118	0.604	0.440
*OXTR*/rs2254298: Avoidance	1	0.56	0.5602	0.553	0.460
Residuals	51	51.62	1.0121		

Number of followers					

Variable	DF	Sum square	Mean square	*F* value	*p*-value

*OXTR*/rs2254298	1	0.009	0.0088	0.082	0.7761
Gender	1	0.650	0.6500	6.035	0.0179 *
Anxiety	1	0.164	0.1637	1.520	0.2241
Avoidance	1	0.034	0.0344	0.319	0.5749
*OXTR*/rs2254298: Gender	1	0.140	0.1400	1.300	0.2603
*OXTR*/rs2254298: Anxiety	1	0.004	0.0036	0.033	0.8566
*OXTR*/rs2254298: Avoidance	1	0.017	0.0167	0.155	0.6953
Gender: Anxiety	1	0.025	0.0253	0.235	0.6305
Gender: Avoidance	1	0.003	0.0033	0.031	0.8608
*OXTR*/rs2254298: Gender: Anxiety	1	0.094	0.0941	0.874	0.3549
*OXTR*/rs2254298: Gender: Avoidance	1	0.041	0.0410	0.381	0.5403
Residuals	45	4.847	0.1077		

**Figure 3:**
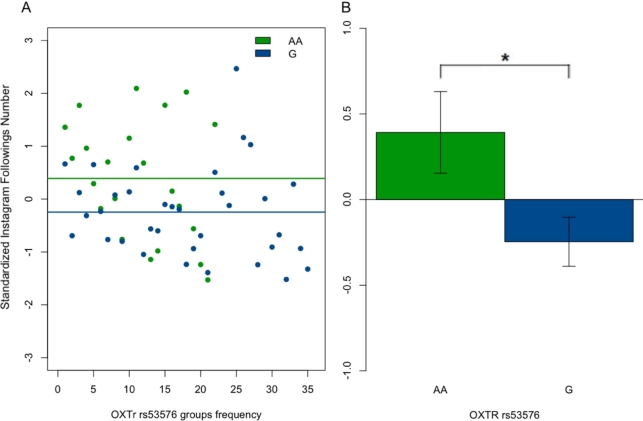
Main Effect of *OXTR*/rs53576 on Instagram Number of Followings. (**A**) Effect of Oxytocin Receptor Gene (*OXTR*) rs53576 on the standardized Instagram number of followings. Green circles = A/A homozygotes; blue circles = G-carriers. Lines constitute the linear models for A/A homozygotes (green) and G-carriers (blue). (**B**) Comparison between the number of Instagram followings in A/A homozygotes (green) and G-carriers (blue) (* *p* < 0.025).

To explore the direction of the ANCOVA effect, a further post-hoc two-tailed Student's *t* test was computed. The Student's *t* test confirmed that the number of Instagram followings was significantly different between the A/A vs G allele groups (*t* = 2.45, df = 55, *p* = 0.018, 95% CI [0.12, 1.16]; see Fig. 3).

Besides the effect of gender on the number of followers, no other significant main effect of *OXTR*/rs53576 or interaction effect between the ECR-R dimensions, gender and *OXTR*/rs53576 were found on Instagram number of followers (*p* > 0.025), as reported in Table 6.

With regards to *OXTR*/rs2254298, main effect of gender on the number of followers apart, no other significant main effect of genotype or interaction effect between the ECR-R subscales, gender and genotype was identified on Instagram number of followings or followers, as reported in Table 6.

### 3.2 Results obtained excluding outliers

#### 3.2.1 Preliminary analysis on the genetic variables

When excluding the outlier values, the genetic distributions varied in relation to the Instagram variable that was considered for the analysis. For *OXTR*/rs53576, the frequencies of genotype were: A/A = 20 (36.36%), A/G = 30 (54.55%), G/G = 5 (9.09%) for the number of posts (Hardy-Weinberg Equilibrium: $X^{2}$ (1) = 1.754, *p* = 0.185, ns).; A/A = 22 (40.00%), A/G = 28 (50.91%), G/G = 5 (9.09%) for SDI (Hardy-Weinberg Equilibrium: $X^{2}$ (1) = 0.869, *p* = 0.351, ns); A/A = 22 (39.28%), A/G = 29 (51.79%), G/G = 5 (8.93%) for the number of followers (Hardy-Weinberg Equilibrium: $X^{2}$ (1) = 1.111, *p* = 0.292, ns); A/A = 20 (37.04%), A/G = 29 (53.70%), G/G = 5 (9.26%) for the number of followings (Hardy-Weinberg Equilibrium: $X^{2}$ (1) = 1.450, 0.228, ns). For *OXTR*/rs53576, the frequencies of genotype were: A/A = 3 (5.46%), G/A = 21 (38.18%), G/G = 31 (56.36%) for the number of posts (Hardy-Weinberg Equilibrium: $X^{2}$ (1) = 0.052, *p* = 0.819, ns); A/A = 4 (7.27%), G/A = 21 (38.18%), G/G = 30 (54.55%) for SDI (Hardy-Weinberg Equilibrium: $X^{2}$ (1) = 0.015, *p* = 0.902, ns); A/A = 4 (7.14%), G/A = 21 (37.50%), G/G = 31 (55.36%) for the number of followers (Hardy-Weinberg Equilibrium: $X^{2}$ (1) = 0.029, *p* = 0.864, ns); A/A = 4 (7.41%), G/A = 21 (38.89%), G/G = 29 (53.70%) for the number of followings (Hardy-Weinberg Equilibrium: $X^{2}$ (1) = 0.005, *p* = 0.941, ns).

#### 3.2.2 Preliminary analysis on ECR-R and Instagram variables

Considering the large amount of outliers that emerged from the previous analysis, such values were therefore removed in relation to the dependent variable of interest. In particular, when considering the number of posts in subsequent analysis, 2 participants were removed for they represented extreme values. The same number of participants was removed when considering the SDI as the dependent variable. Conversely, 3 and 1 participants were removed when considering the number of followings and followers respectively as dependent variables. Table 7 reports skewness, kurtosis, and descriptive statistics for the continuous variables of the study.

**Table 7:** Summary of the descriptive statistics for each continuous variable after removing the outliers (N = 2 for Instagram posts; N = 2 for Social Desirability Index; N = 3 for Instagram followings; N = 1 for Instagram followers) from the data pool. The distribution of each ECR-R (Experience in Close Relationships-Revised) dimension and Instagram variable is described in terms of Skewness, Kurtosis, Minimum (Min), first Quartile (1st Q), Median, Mean, third Quartile (3rd Q), Maximum (Max) and Standard Deviation (SD). Instagram variables were standardized using z-score transformation and inspected for normality. Skewness and kurtosis values were computed for each continuous subscale of the ECR-R questionnaire and each Instagram variable. Of all variables, only participants' number of posts did not follow the Gaussian distribution, and was thus adjusted by log-transformation. Log-transformed number of posts shows increased values compared to the original sampling of the same variable.

Variable	Skewness	Kurtosis	Min	1st Q	Median	Mean	3rd Q	Max	SD
Anxiety	-0.38	-0.02	1.39	3.33	3.94	3.94	4.56	6.22	1.08
Avoidance	-0.15	-0.39	1.06	2.11	2.94	2.95	3.67	5.39	0.93
Posts number	1.53	1.64	-0.60	-0.52	-0.39	-0.16	0.01	1.36	0.50
Log-transformed posts number	1.14	0.33	0.33	0.39	0.48	0.58	0.70	1.21	0.24
Social Desirability Index	-0.69	2.45	-1.16	-0.28	-0.13	-0.16	-0.01	0.61	0.30
Followings	0.34	-0.74	-1.53	-0.79	-0.14	-0.12	0.57	1.78	0.89
Followers	0.77	0.11	-0.80	-0.51	-0.17	-0.11	0.22	1.45	0.51

Again, four preliminary two-tailed Student's *t*-tests (corrected α=0.0125) were computed to examine the existence of a gender effect on the Instagram variables. No significant difference between males and females participants' scores was detected for any of the Instagram variables (number of posts: *t* = 0.68, df = 53, *p* = 0.498, 95% CI [-0.09, 0.19]; SDI: *t* = 0.90, df = 55, *p* = 0.374, 95% CI [-0.10, 0.26]; number of followings: *t* = 2.53, df = 52, *p* = 0.015, 95% CI [0.13, 1.15]; number of followers: *t* = 1.84, df = 54, *p* = 0.071, 95% CI [-0.02, 0.58]). Thus, participants' gender was not included in the subsequent analysis as a moderator variable.

#### 3.2.3 Hypothesis-driven analysis: Instagram number of posts and SDI

Means and standard error means of Instagram number of posts and SDI for *OXTR*/rs53576 and *OXTR*/rs2254298 are reported in Table 8.

**Table 8:** Mean values in Oxytocin Receptor Gene (*OXTR*) rs53576 A/A homozygotes and G-carriers and Oxytocin Receptor Gene (*OXTR*) rs2254298 G/G homozygotes and A-carriers divided by Experience in Close Relationships-Revised (ECR-R) dimensions (high or low) on Instagram number of posts and Social Desirability Index, after removing the outlier values. Standard error means are reported between parentheses. Instagram behavior is reported by users' *OXTR*/rs53576 phenotype and ECR-R dimensions, consistently with the literature on the topic and a previous publication [60].

*OXTR*/rs53576				

Number of posts				

ECR-R dimension	Low/AA	Low/G	High/AA	High/G
Anxiety	0.66 (0.07)	0.54 (0.05)	0.54 (0.07)	0.58 (0.07)
Avoidance	0.71 (0.08)	0.61 (0.06)	0.49 (0.04)	0.51 (0.06)

Social desirability index				

ECR-R dimension	Low/AA	Low/G	High/AA	High/G
Anxiety	-0.20 (0.05)	-0.04 (0.10)	-0.13 (0.04)	-0.26 (0.08)
Avoidance	-0.13 (0.04)	-0.12 (0.10)	-0.20 (0.04)	-0.20 (0.09)

*OXTR*/rs2254298				

Number of posts				

ECR-R dimension	Low/GG	Low/A	High/GG	High/A
Anxiety	0.60 (0.06)	0.56 (0.07)	0.59 (0.07)	0.54 (0.07)
Avoidance	0.67 (0.06)	0.61 (0.06)	0.52 (0.05)	0.49 (0.07)

Social desirability index				

ECR-R dimension	Low/GG	Low/A	High/GG	High/A
Anxiety	-0.14 (0.08)	-0.06 (0.10)	-0.22 (0.08)	-0.21 (0.06)
Avoidance	-0.14 (0.06)	-0.11 (0.11)	-0.23 (0.10)	-0.16 (0.04)

As in the statistical plan of the study, Equation (1) was employed to test the existence of potential gene*environment interactions on Instagram number of posts and SDI. These hypothesis-driven analyses did not result in any main effect on the Instagram number of posts or SDI. Moreover, no interaction effect emerged between the SNP of interest (either *OXTR*/rs53576 or *OXTR*/rs2254298) and the two attachment dimensions (see Table 9).

**Table 9:** Hypothesis-driven analysis: statistics' summary of ANCOVAs on Instagram number of posts and Social Desirability Index. For each Instagram variable considered in the hypothesis (Instagram number of posts and SDI) as dependent variable, one mixed ANCOVA was performed with *OXTR* gene genotype rs53576 (A/A and G-carriers) as a between-subject factor and the ECR-R dimensions (Anxiety and Avoidance) as continuous covariates (see the top section of the Table). For each Instagram variable, one mixed ANCOVA was also performed with the *OXTR* gene genotype rs2254298 (G/G and A-carriers) as a between-subject factor and the other parameters unchanged (see the below section of the Table).

*OXTR*/rs53576					

Number of posts					

Variable	DF	Sum square	Mean square	*F* value	*p*-value

*OXTR*/rs53576	1	0.0229	0.02292	0.429	0.5156
Anxiety	1	0.0131	0.01314	0.246	0.6223
Avoidance	1	0.1682	0.16820	3.147	0.0823
*OXTR*/rs53576: Anxiety	1	0.2006	0.20064	3.754	0.0584
*OXTR*/rs53576: Avoidance	1	0.0075	0.00749	0.140	0.7097
Residuals	49	2.6187	0.05344		

Social desirability index					

Variable	DF	Sum square	Mean square	*F* value	*p*-value

*OXTR*/rs53576	1	0.000	0.00027	0.003	0.957
Anxiety	1	0.163	0.16284	1.772	0.189
Avoidance	1	0.012	0.01170	0.127	0.723
*OXTR*/rs53576: Anxiety	1	0.108	0.10788	1.174	0.284
*OXTR*/rs53576: Avoidance	1	0.000	0.00001	0.000	0.993
Residuals	49	4.504	0.09192		

*OXTR*/rs2254298					

Number of posts					

Variable	DF	Sum square	Mean square	*F* value	*p*-value

*OXTR*/rs2254298	1	0.0284	0.02835	0.500	0.4828
Anxiety	1	0.0080	0.00804	0.142	0.7082
Avoidance	1	0.1834	0.18339	3.235	0.0782
*OXTR*/rs2254298: Anxiety	1	0.0116	0.01157	0.204	0.6534
*OXTR*/rs2254298: Avoidance	1	0.0221	0.02208	0.390	0.5354
Residuals	49	2.7777	0.05669		

Social desirability index					

Variable	DF	Sum square	Mean square	*F* value	*p*-value

*OXTR*/rs2254298	1	0.024	0.02375	0.261	0.612
Anxiety	1	0.155	0.15462	1.702	0.198
Avoidance	1	0.009	0.00856	0.094	0.760
*OXTR*/rs2254298: Anxiety	1	0.033	0.03327	0.366	0.548
*OXTR*/rs2254298: Avoidance	1	0.114	0.11382	1.253	0.269
Residuals	49	4.453	0.09087		

#### 3.2.4 Exploratory analysis: Instagram number of followings and followers

Means and standard error means of Instagram number of followings and followers for *OXTR*/rs53576 and *OXTR*/rs2254298 are reported in Table 10.

**Table 10:** Mean values in Oxytocin Receptor Gene (*OXTR*) rs53576 A/A homozygotes and G-carriers and Oxytocin Receptor Gene (*OXTR*) rs2254298 G/G homozygotes and A-carriers divided by Experience in Close Relationships-Revised (ECR-R) dimensions (high or low) on Instagram number of followings and followers, after removing the outlier values. Standard error means are indicated between parentheses. Instagram behavior is reported by users' *OXTR*/rs2254298 phenotype and ECR-R dimensions, consistently with the literature on the topic and a previous publication [60].

*OXTR*/rs53576				

Number of followings				

ECR-R dimension	Low/AA	Low/G	High/AA	High/G
Anxiety	0.53 (0.30)	-0.23 (0.19)	-0.08 (0.34)	-0.42 (0.15)
Avoidance	0.54 (0.31)	-0.20 (0.20)	-0.25 (0.28)	-0.44 (0.14)

Number of followers				

ECR-R dimension	Low/AA	Low/G	High/AA	High/G
Anxiety	0.18 (0.14)	-0.16 (0.12)	-0.14 (0.16)	-0.26 (0.12)
Avoidance	0.13 (0.15)	-0.07 (0.14)	-0.07 (0.16)	-0.35 (0.07)

*OXTR*/rs2254298				

Number of followings				

ECR-R dimension	Low/GG	Low/A	High/GG	High/A
Anxiety	-0.10 (0.23)	0.23 (0.27)	-0.40 (0.22)	-0.18 (0.23)
Avoidance	-0.07 (0.23)	0.37 (0.31)	-0.46 (0.21)	-0.31 (0.16

Number of followers				

ECR-R dimension	Low/GG	Low/A	High/GG	High/A
Anxiety	-0.05 (0.13)	0.04 (0.14)	-0.20 (0.16)	-0.24 (0.10)
Avoidance	-0.03 (0.15)	0.08 (0.15)	-0.23 (0.14)	-0.28 (0.07)

From the performed ANCOVAs, a main effect of the *OXTR*/rs53576 on the number of followings on Instagram was observed (*F*(1, 53) = 5.78, *p* = 0.020, p$\eta^{2}$ = 0.11). No other main effect of the two SNPs or ECR-R dimensions, and neither for their interplay, was observed on the Instagram variables (see Table 11).

**Table 11:** Exploratory analysis: statistics' summary of ANCOVAs on Instagram number of followings and number of followers. For each Instagram variable considered in the exploratory analysis (Instagram number of followings and followers) as dependent variable (DV), one mixed ANCOVA was performed with *OXTR* gene genotype rs53576 (A/A and G-carriers) as a between-subject factor and the ECR-R dimensions (Anxiety and Avoidance) as continuous covariates, as in Equation (1) (see the top section of the Table). For each Instagram variable, one mixed ANCOVA was also performed with the *OXTR* gene genotype rs2254298 (G/G and A-carriers) as a between-subject factor and the other parameters unchanged (see the below section of the Table). A significant main effects of the genotype was found on the number of followings for *OXTR*/rs53576. (* *p* < 0.025).

*OXTR*/rs53576					

Number of followings					

Variable	DF	Sum square	Mean square	*F* value	*p*-value

*OXTR*/rs53576	1	3.83	3.831	5.776	0.0202 *
Anxiety	1	1.42	1.416	2.135	0.1505
Avoidance	1	2.40	2.398	3.617	0.0632
*OXTR*/rs53576: Anxiety	1	1.03	1.034	1.560	0.2178
*OXTR*/rs53576: Avoidance	1	0.13	0.130	0.197	0.6595
Residuals	48	31.83	0.663		

Number of followers					

Variable	DF	Sum square	Mean square	*F* value	*p*-value

*OXTR*/rs53576	1	0.835	0.8346	3.378	0.072
Anxiety	1	0.398	0.3981	1.611	0.210
Avoidance	1	0.398	0.3979	1.610	0.210
*OXTR*/rs53576: Anxiety	1	0.168	0.1680	0.680	0.414
*OXTR*/rs53576: Avoidance	1	0.139	0.1395	0.565	0.456
Residuals	50	12.354	0.2471		

*OXTR*/rs2254298					

Number of followings					

Variable	DF	Sum square	Mean square	*F* value	*p*-value

*OXTR*/rs2254298	1	0.91	0.906	1.273	0.2649
Anxiety	1	1.78	1.784	2.506	0.1200
Avoidance	1	3.51	3.507	4.928	0.0312
*OXTR*/rs2254298: Anxiety	1	0.05	0.050	0.070	0.7920
*OXTR*/rs2254298: Avoidance	1	0.23	0.234	0.329	0.5692
Residuals	48	34.16	0.712		

Number of followers					

Variable	DF	Sum square	Mean square	*F* value	*p*-value

*OXTR*/rs2254298	1	0.003	0.0031	0.012	0.914
Anxiety	1	0.537	0.5369	2.040	0.159
Avoidance	1	0.534	0.5336	2.028	0.161
*OXTR*/rs2254298: Anxiety	1	0.036	0.0362	0.138	0.712
*OXTR*/rs2254298: Avoidance	1	0.026	0.0258	0.098	0.755
Residuals	50	13.156	0.2631		

**Figure 4:**
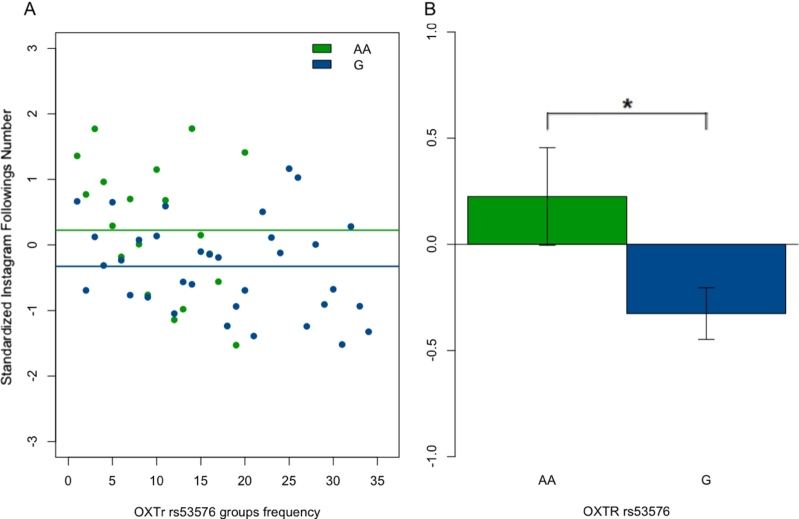
Main Effect of *OXTR*/rs53576 on Instagram Number of Followings. (**A**) Effect of Oxytocin Receptor Gene (*OXTR*) rs53576 on the standardized Instagram number of followings. Green circles = A/A homozygotes; blue circles = G-carriers. Lines constitute the linear models for A/A homozygotes (green) and G-carriers (blue). (**B**) Comparison between the number of Instagram followings in A/A homozygotes (green) and G-carriers (blue) (* *p* < 0.025).

As before, the direction of the observed effect was studied with a post-hoc two-tailed Student's *t* test. In particular, a significant difference between allelic groups (A/A vs. G alleles) on the number of followings was confirmed (*t* = 2.33, df = 52, *p* = 0.024, 95% CI [0.08, 1.03]; see Fig. 4).

## 4 Discussion

For the more rigorous analyses applied, the discussion will focus on the results obtained excluding outliers. Two gene*environment interactions on the number of posts and SDI (*OXTR*/rs53576 SNP * close relationship in adulthood; *OXTR*/rs2254298 SNP * close relationship in adulthood) were initially hypothesized. Contrary to these predictions (see the section “Limitations” for an extensive overview on the expected patterns and the obtained results), only a main effect of gene *OXTR*/rs53576 on Instagram number of followings was found. Post-hoc tests revealed differential Instagram behavior between the two genetic groups within the *OXTR*/rs53576. Independent of the reported scores on anxiety and avoidance in adult relationships, A/A homozygotes showed a higher number of followings compared to G-carriers. Previous researches highlighted that *OXTR*/rs53576 G allele is linked to higher marital satisfaction, support seeking in hostile circumstances, and higher empathy than A-carriers [55], [96]. Conversely, in a few cases, A/A individuals were found to display increased social attitudes and higher social sensitivity than G-carriers [97]. On the other hand, the G allele of the *OXTR*/rs2254298 has been associated with separation anxiety, depression, decreased online sociability and lower reciprocity in a support-giving interaction [56], [61], [98]. Overall, the role played by the A and G alleles on the *OXTR* SNPs towards general social behavior is debated [53]. This debate will be clearer if the genetic correlates of social media behavior were investigated.

In connection to the findings of the study, distinct biological roots could have differently shaped online behavior among genetic groups (i.e. *OXTR*/rs53576 A/A versus G-carriers), specifically the level of online sociability on SNSs, like Instagram. A high number of followings could represent the desire of Instagram's user to be involved in social interactions [61], [99]. Specifically, A/A homozygotes could exhibit a genetically-founded inclination to online sociability witnessed by a higher frequency of online behaviors (i.e. Instagram number of followings) than G-carriers. As a protective factor [100], [101], A allele could predispose the user to be less anxious in online prosocial activities and proactively seek contact with other Instagram users. In contrast, G allele could predispose the user to be less interested or less motivated to search for others on Instagram. Given the contradictory results from the literature on the alleles of *OXTR*/rs53576 [102], [103], [104], it is open to question which allele is the bearer of a detrimental effect. However, in light of the *Differential Susceptibility model* [105], we assumed that the genetic properties related to a given allele could not wholly determine online sociability only in terms of the “positive” (protective factor) versus “negative” (risk factor) predisposition [106]. Rather, every individual could exhibit a range of virtual social behaviors as a function of the interaction between genetic sensitivity to experiences, and environmental influences [107], [108]. Within this framework, the current results suggest that no interaction between the quality of close adult relationships and the genetic predispositions for *OXTR*/rs53576 or *OXTR*/rs2254298 could explain Instagram sociability under a gene*environment perspective. This outcome finds agreement with previous research which did not identify an association between the *OXTR*/rs53576 and close relationship main dimensions [109], [110]. In contrast to the present results, a previous work [60] revealed that the interaction between *OXTR*/rs2254298 and the early parental bonding could modulate the Instagram number of posts and SDI. Nevertheless, the authors did not detect any gene*environment effect for *OXTR*/rs53576 on Instagram sociability. In conclusion, multiple environmental components (i.e. quality of relationships with people) and genetic factors (i.e., *OXTR*/rs53576 or *OXTR*/rs2254298) could differently modulate users' online prosociality and frequency of virtual interactions (i.e. high number of followings). Since no interaction effect between genotype and close relationships was found, here, *OXTR*/rs53576 could shape the level of Instagram sociability independent of the potential influence of the close adult relationship with one's partner. The current findings could inspire future research in exploring online sociability under a gene-environment perspective.

## 5 Limitations

### 5.1 A genetic overview

One specific hypothesis was formulated a-priori: *OXTR*/rs224529 A-carriers and *OXTR*/rs53576 G-carriers Instagram users would show a differential number of posts and values of SDI in relation to the quality of relationship with the partner (a higher number of posts and values of SDI when low scores in anxiety and avoidance are reported; a lower number of posts and values of SDI when high scores in anxiety and avoidance are reported) compared to *OXTR*/rs53576 A/A homozygotes and *OXTR*/rs224529 G/G homozygotes users. To verify our hypothesis, multiple mixed ANCOVAs have been employed. The existence of an interaction effect with the predicted directionality would have confirmed our hypotheses. Contrary to our expectations, no significant interaction effects were found. In addition to the ANCOVAs employed to verify our hypothesis, exploratory analyses were performed. Contrary to the results of our hypothesis-driven tests, the exploratory analysis revealed a main effect of the gene *OXTR*/rs53576 on Instagram number of followings. Within the *OXTR*/rs53576, post-hoc tests confirmed a significant difference of Instagram behavior between A/A and G-carriers. Independent of the anxiety and avoidance felt towards the partner, A/A homozygotes showed a higher number of followings compared to G-carriers. At a genetic level, the discrepancy between the expected patterns and the obtained results requires some considerations. For instance, an absolute consensus on the predisposition given by *OXTR*/rs53576 A or G allele does not exist [111] and the culture-dependent variation of allelic distributions should not be ignored [112]. Nonetheless, no interaction effect was detected between genes and close relationship scores. This finding is theoretically valid and confirmed by previous researches which highlighted no associations between *OXTR* and adult attachment [113], [114], [115]. However, a potential issue in this study could be traced back to the limited sample size, a condition that is likely to determine that power values were not sufficient to obtain the hypothesized effect. Moreover, no results were found for the region *OXTR*/rs2254298, which was previously found to be highly involved in general sociability [116]. Overall, the present results cannot unequivocally define the role played by *OXTR*/rs53576 and *OXTR*/rs224529 on social media behavior.

### 5.2 Methodological limitations

Alongside the aforementioned genetic considerations, other methodological limitations need to be discussed. Firstly, the sample size was small (N = 57) with a substantial prevalence of female participants. Secondly, Instagram data were not adjusted by the participants' time of membership. Although Instagram data were extracted at the same time-point, the current study does not take into account the year of the first registration for each Instagram user or the evolution of the online social activity across time (e.g., in terms of short-term daily usage and long-term increased number of posts, followings and followers). While this would have been an interesting variable to include in our analysis, this information is not publicly available, and therefore not acquirable with our method. Obtaining access to this information could allow to correct the number of posts, followers, and followings by the amount of time passed between the date of creation of an account and the instant in which data were extracted. Future works should consider obtaining this information in order to obtain a more standardized set of variables. Thirdly, the obtained results cannot be generalized to online sociability which exist outside Instagram. The activity of alternative social network sites like Facebook and Twitter should be monitored to discover similar or dissimilar patterns of online socialization. Moreover, the current investigation did not collect Instagram data from the participants' partners. Being aware of Instagram attitudes on both participant and the partner would allow for comparing the quality in the intimate relationship and the frequency of social media behavior in terms of posts, followings, followers and SDI. Participants were not asked to report elements on the quality of their relationship with Instagram followings. Furthermore, participants were not questioned to indicate how many followings and followers they know and how often they meet them in daily life. Finally, important information may be provided by dispositional characteristics such as personality traits and “real-life” social environments that cannot be accounted by the partnership. It is worth noting that the same housing conditions of the participants and their availability of technological devices could alter their access to Instagram.

## 6 Conclusions

Overall, the present results should be interpreted with a great degree of caution in the panorama of genetic association studies [117], [118], [119]. Data interpretation is even more challenging in the presence of a genetic influence independent of the environmental exposure [120], [121] on an underexplored social variable such as Instagram number of followings. Although there is no strong evidence of gene-environment interactions on online sociability, the existing literature on general or in-person sociability or emotional support reveals that the allelic distribution varies across ethnicities and multiple factors could determine the role that A or G alleles play in the individuals' lives [122]. For instance, several studies tested the socio-emotional behavior and distress explained by variations in *OXTR*/rs53576 [123], [124], [125] or by its potential interaction with attachment [110] on a collectivist culture sample. The same differential social responsibility towards other Instagram users between A/A homozygotes and G-carriers could also depend on baseline differences, such as personality traits, ethnicity, vulnerability to psychiatric disorders [126]. Altogether, these factors could similarly have implicitly modulated the online sociability of the participants. A provocative question will be if *OXTR*/rs53576 A and G allele maintain the same influence on physical (i.e. in-person social interaction) and virtual environments (i.e. online sociability on Instagram).

Future studies should measure such factors and control their impact on Instagram behavior to control potential biases. Moreover, future works should increase the sample size and explore close relationships through alternative questionnaires, such as self-reported measures or observational techniques (e.g. using the Adult Attachment Interview [127]). Finally, consider the different genetic regions involved in the modulation of other neurotransmitters (e.g. 5-HTTLPR and DRD4) as potential factors of predisposition.

These preliminary and exploratory results are shared with the scientific community to refine future research in the field.

## Declarations

### Author contribution statement

Alessandro Carollo, Andrea Bonassi: Analyzed and interpreted the data; Contributed analysis tools or data; Wrote the paper.

Ilaria Cataldo: Conceived and designed the experiments; Performed the experiments; Wrote the paper.

Giulio Gabrieli: Analyzed and interpreted the data; Wrote the paper.

Moses Tandiono, Jia Nee Foo: Contributed reagents, materials, analysis tools or data.

Bruno Lepri, Gianluca Esposito: Conceived and designed the experiments; Wrote the paper.

### Funding statement

This research was supported by grants from the NAP SUG (M4081597, 2015-2021) and the Ministry of Education, Singapore, under its Academic Research Fund Tier 1 (RG55/18) to G.E.

### Data availability statement

Data associated with this study has been deposited at NTU's Data repository (DR NTU Data) under the url: https://doi.org/10.21979/N9/GUSTLQ.

### Declaration of interests statement

The authors declare no conflict of interest.

### Additional information

No additional information is available for this paper.
